# Organoids model distinct Vitamin E effects at different stages of prostate cancer evolution

**DOI:** 10.1038/s41598-017-16459-2

**Published:** 2017-11-24

**Authors:** Rose N. Njoroge, Kenji Unno, Jonathan C. Zhao, Anum F. Naseem, Jonathan F. Anker, Warren A. McGee, Larisa Nonn, Sarki A. Abdulkadir

**Affiliations:** 1 Northwestern University, Feinberg School of Medicine, Department of Urology, Chicago, IL 60611 USA; 2 Division of Hematology/Oncology, Department of Medicine, Northwestern University Feinberg School of Medicine, Chicago, IL USA; 3 Department of Neurology, Northwestern University, Chicago, IL USA; 4 Department of Pathology, University of Illinois at Chicago, Chicago, IL 60612 USA; 5 Robert H. Lurie Comprehensive Cancer Center, Northwestern University Feinberg School of Medicine, Chicago, IL USA; 6 Department of Pathology, Northwestern University Feinberg School of Medicine, Chicago, IL USA

## Abstract

Vitamin E increased prostate cancer risk in the Selenium and Vitamin E Cancer Prevention Trial (SELECT) through unknown mechanisms while Selenium showed no efficacy. We determined the effects of the SELECT supplements on benign (primary), premalignant ( RWPE-1) and malignant (LNCaP) prostate epithelial organoids. While the supplements decreased proliferation and induced cell death in cancer organoids, they had no effect on the benign organoids. In contrast, Vitamin E enhanced cell proliferation and survival in the premalignant organoids in a manner that recapitulated the SELECT results. Indeed, while Vitamin E induced a pro-proliferative gene expression signature, Selenium alone or combined with Vitamin E produced an anti-proliferative signature. The premalignant organoids also displayed significant downregulation of glucose transporter and glycolytic gene expression pointing to metabolic alterations. Detached RWPE-1 cells had low ATP levels due to diminished glucose uptake and glycolysis which was rescued by Vitamin E through the activation of fatty acid oxidation (FAO). FAO inhibition abrogated the ATP rescue, diminished survival of the inner matrix detached cells, restoring the normal hollow lumen morphology in Vitamin E treated organoids. Organoid models therefore clarify the paradoxical findings from SELECT and demonstrate that Vitamin E promotes tumorigenesis in the early stages of prostate cancer evolution.

## Introduction

Prostate cancer (PCa) is the most commonly diagnosed non-skin cancer in American men with 161,360 new cases predicted in 2017 alone^1^. High disease incidence coupled with a lack of effective treatments make PCa prevention a public health priority. Antioxidants are potentially chemopreventive against cancer as they scavenge ROS preventing DNA damage and genome instability^2^. Indeed, preclinical studies and the secondary analyses of the Nutritional Prevention of Cancer (NPC) and the Alpha-Tocopherol Beta Carotene (ATBC) clinical trials indicated that the antioxidants Vitamin E and Selenium might protect against PCa^3,4^. However, a subsequent large scale PCa chemoprevention study (n = 35,533), the Selenium and Vitamin E Cancer Prevention Trial (SELECT), found no benefit for Selenium and an unexpected 17% increase in PCa risk in the Vitamin E arm^5^.

The outcome of the SELECT trial remains a puzzle. Some attribute the lack of efficacy to the doses and formulations of Vitamin E/Selenium used^6,7^. SELECT tested a daily dose of Vitamin E, (α-tocopheryl acetate; 400 mg), and/or Selenium (L-selenomethionine; 200 μg)^8^. The ATBC trial had however demonstrated efficacy with a much lower dose of 50 mg α-tocopheryl acetate while the NPC trial had used 200 μg Selenized yeast^3,4^. Furthermore, Vitamin-E isomers have different bioactivities; due to its ability to scavenge both ROS and reactive nitrogen species (RNS), γ-tocopherol is superior to α-tocopherol^9^. Finally, while SELECT was an intervention on healthy men, a different study suggested that Vitamin E might be effective against advanced but not latent or early PCa^10^. We hypothesized that prostate cells at different stages of the cancer evolution process may respond differently to antioxidants.

While conventional two-dimensional (2D) tissue culture has been useful in unravelling the biology of prostate cancer, important limitations restrict its utility. Cells in 2D lack physiological cell and matrix interactions and attachment to artificial surfaces affects cell morphology and signaling. Additionally, lack of oxygen and nutrient gradients in 2D cultures makes the environment non-physiologically uniform^11^. The use of animal models like the genetically engineered (GEM), xenograft and tissue recombination mouse models overcomes some of these limitations. However, systemic and physiologic differences between mouse and human prostates can affect phenotype^12^. GEM models are expensive and also take long to generate while xenograft models are limited by the few number of available prostate cancer cell lines^12^. Moreover, the intractability of whole animal models makes them less ideal for investigating molecular mechanisms at the cellular level necessitating cell cultures^13^.

In three dimensional (3D) cultures, cells form cell-cell and cell-matrix attachments mimicking an *in-vivo* environment^14^. Additionally; growth factor, nutrients and oxygen gradients in 3D cultures yield heterogeneous cell populations like *in-vivo* ^14^. Due to the shortcomings of 2D cell culture^15^ and the difficulty of modeling the spectrum of human prostate tumorigenesis *in vivo*, we turned to organoid culture. We modeled different stages of PCa progression in 3D organoids and tested the effects of the SELECT supplements on PCa tumorigenesis.

## Methods

### Cell culture

Benign human primary prostate epithelial cells were acquired after approval from the University of Illinois Institutional Review Board (IRB) and informed consent obtained as previously described^16^. Experiments on the human samples were performed in accordance with stipulated guidelines and regulations. The primary cells were expanded once in PrEGM media (Lonza no. CC-3166) prior to organoid culture. LNCaP cells were grown in RPMI 1640 media (Gibco Life Technologies no. 11875-093) supplemented with 10% fetal bovine serum (FBS) - (Life Technologies no. 10437-028) and 1% penicillin/streptomycin antibiotic solution (Life Technologies no. 15140-122). RWPE-1 cells were grown in keratinocyte serum-free media supplemented with 0.05 mg/ml bovine pituitary extract, 5 ng/ml epidermal growth factor (Thermo Fischer Scientific no. 17005042) and 1% penicillin/streptomycin antibiotic solution. All cells were mycoplasma free and cell lines genetically authenticated.

### 2D Cell Proliferation Assay

To monitor the effect of the SELECT supplements on cell proliferation in 2D, replicates of pretreated RWPE-1 (n = 6–9) were plated in 96-well plates at densities of 2000 cells/well. The plates were scanned with the IncuCyte ZOOM^TM^ live cell imaging system (Essen BioScience) with continued treatments. Images were captured every four hours for the durations indicated using the 10x objective. Cell confluence was calculated with the IncuCyte ZOOM^TM^ software (version 2015A).

### Next-Generation Sequencing (NGS)

A customized NGS panel targeting 222 cancer related genes was used to sequence DNA. DNA Probes for capturing exon regions in these genes were manufactured by Roche NimbleGen. SeqCap EZ Library SR User’s Guide (Roche, Pleasanton, CA) was followed for library preparation and capture of targeted sequences. Paired-end sequencing of 2 × 150 bp was performed on a MiSeq platform (Illumina, San Diego, USA). Twelve individual libraries were multiplexed for a MiSeq flow cell. The mean sequencing depth of coverage was 135x.

### Bioinformatics analysis

Paired-end reads were aligned to the GRCh37 version of the human genome using Burrows-Wheeler Aligner v0.7 to generate BAM files^17^. After sorting the BAM files using samtools, PCR duplicates marked using Picard and realignment around putative gaps was performed using the Genome Analysis Toolkit (GATK) v3.2-2. Variant calling was performed with the GATK Haplotype caller. ANNOVAR (http://annovar.openbioinformatics.org/en/latest) was used for annotating variants and for retrieving information on variants in the population-based studies such as the 1000 Genomes Project (www.1000genomes.org), NHLBI-ESP 6500 exomes or ExAC (http://exac.broadinstitute.org/), and clinical databases such as the Human Gene Mutation Database (HGMD)^18^ and ClinVar^19^. Pathogenicity of variants is defined based on American College of Medical Genetics and Genomics (ACMG) criteria^20^. Specifically, pathogenic and likely pathogenic mutations are defined as (1) all protein truncating mutations unless their allele frequency is 5% or higher in any racial group in population databases or is reported as benign or likely benign in the ClinVar, and (2) nonsynonymous changes if their allele frequency is less than 5% and reported as pathogenic and likely pathogenic mutations in the ClinVar. The NGS sequencing and analysis were carried out by the Genomics Core Facility at the NorthShore University Health System (Chicago, IL).

### Organoid culture and treatments

After expansion in their optimum media, the different cell types were trypsinized and transferred to organoid media as described by Unno *et al*.^16^. Briefly; 5000 cells were resuspended in organoid media containing low percentage matrigel (5%) then plated in to 96-well ultralow attachment plates (Corning no. 3474). A hunderd microliters of fresh media was added to the cultures every four days or every two days once the treatments commenced. Treatments were added to the following final concentrations; 40 μM DL-αTocopherol-Acetate (Sigma no. T3376), 40 μM RRR-γ-Tocopherol (Sigma no. T1782) and/or 1.3 μM Seleno-L-methionine (Sigma no. S3132) representing the mean concetrations attained in the blood plasma of the SELECT subjects^21^. N-acetyl cysteine (NAC; Sigma no. A9165) and Etomoxir sodium salt hydrate (Eto; Sigma no. E1905) were used at various concentrations as indicated in the figures. Organoid growth was captured by brightfield microscopy using Zeiss Axioskop/Nuance microscope (Carl Zeiss Inc. Oberkochen, Germany).

### Histology and immunostaining

Fixation, processing, H&E and immunofluorescence staining were done as previously described^16^. The following primary antibodies were used: Ki-67 (1:100; eBioscience no.14-5698-80), BrDU (1:100; Abcam no. ab6326), CK8 (1:400; Covance no. MMS-162P) and CK14 (1:500; Covance no. PRB-155P). The secondary antibodies (Life Technologies) used at 1:400 each were; goat anti-rabbit Alexa Fluor 647 (no. A21244), goat anti-rat Alexa Fluor 488 (no. A11006) and goat anti-mouse Alexa Fluor 568 (no. A11004). Sections were counterstained with 0.5 mg/ml DAPI (Sigma no. D-9542) and mounted in ProLong Diamond Antifade reagent (Molecular Probes no. P36961). When appropriate, BrDU (Invitrogen no. 00-0103) was added into the organoid medium at 1:100 dilution (3 μg/mL) overnight. BrdU was detected using a rat anti-BrdU antibody (1:100; Abcam no. ab6326). Imaging was performed using a Nikon A1R (A) Spectral laser scanning confocal microscopy (Nikon Instruments Inc. Yokohama, Japan, Japan).

### Detection of Reactive Oxygen Species (ROS)

Mitochodrial ROS were detected using CellROX Green (Thermo Scientific C10444) according to the manufacturer’s instructions. In brief; on day 21 of organoid culture, the probe was added to a final probe concentration of 5 μM. Staining was done in the dark for 1 hour at 37 °C. Organoids were washed in PBS and placed in chamber slides for imaging using the Nikon A1R (A) Spectral laser confocal microscope. The mean fluorescence intensity per image was determined using the Fiji (ImageJ) software.

### Microarray analysis of antioxidant treated organoids

RNA was extracted using Trizol (Life Technologies No. 15596-026) from triplicates of organoids pooled from several 96-plate wells. The RNA was cleaned up using an RNeasy Mini Kit (Qiagen no. 74104) and DNAse (Qiagen no. 79254) on column treatment. The RNA was hybridized to Affymetrix HTA 2.0 transcriptome arrays and analyzed with the Affymetrix AGCC suite at the University of Illinois at Chicago (UIC) Genomics core (Chicago, IL). The CEL files were imported in to R (windows version 3.1.1) using the Bioconductor (version 3.3) oligo package. Raw intensity scores for probes were normalized by quantiles and background corrected with RMA. Differentially expressed genes (DEGs) were identified by the Bioconductor limma package. For functional analysis, the C2 (curated) gene sets of MSigDB (version 5.1) were queried using the Genome Set Enrichment Analysis (GSEA) algorithm^22^. Results were visualized with the Enrichment Map plug-in^23^ [version 2.0.1] on Cytoscape^24^ [version 3.2.1] using a p-value cutoff of 0.005, an FDR cutoff of 0.25, and an overlap coefficient cutoff of 0.5.

### Glucose absorption assay

We plated 11,000 cells per well in 96-well plates with or without a 1.5% poly-HEMA coating (Sigma no. P3932). Media was collected after 24 h and diluted 1:2000 in water. The amount of glucose was measured using the Amplex Red Glucose Assay Kit (Thermo Fisher Scientific no. A22189) per the manufacturer’s instructions.

### ATP assay

Cells were plated in poly-HEMA coated or uncoated 96-well plates at a density of 11,000 cells per well. After 24 h, ATP was measured using the ATPlite Luminescence kit (PerkinElmer no. 6016943) per the manufacturer’s protocol.

### Oxygen consumption (OCR) and extracellular acidification rate (ECAR) measurements

To measure the effects of the SELECT supplements on the bioenergetics of RWPE-1 cells, treatments were done for 5 days before switching them to non-adherent conditions for 24 hours. Cells were plated in replicates (n = 15) at a density of 30,000 cells/well in the XF96 well Seahorse cell culture plates (Agilent no. 101085-004) coated with Celltak (Corning no. 354240). Oxygen consumption and extracellular acidification rates were measured in a XF96 extracellular flux analyzer (Seahorse Bioscience). Oligomycin, Carbonyl cyanide 3-chlorophenylhydrazone (CCCP), Antimycin, Rotenone and 2-Deoxy-D-glucose (2DG) (Sigma) were injected to final concentrations of 5 µM, 0.75 µM, 2 µM, 2 µM, and 40 mM respectively. Experiments were performed in the DMEM based XF Base medium (Agilent Technologies no. 103335-100) without phenol red, bicarbonate, glucose or glutamine. The medium was supplemented with 10 mM glucose (Sigma), 2 mM glutamine (Sigma), 5 mM HEPES (Sigma no. H0887), 2.5 µg human recombinant Epidermal Growth Factor and 25 mg Bovine Pituitary Extract (BPE) (Thermo Fisher Scientific no. 17005042). Basal OCR is the OCR value before the injection of any drugs and after the subtraction of the OCR values after the injection of antimycin A and rotenone (A/R) to discount non-mitochondrial OCR. Maximal OCR is the OCR value after the induction of respiration with CCCP subtracting the non-mitochondrial OCR. Basal and maximal ECAR are the sensitivity of the extracellular acidification rate before the injection of any drugs and the injection of oligomycin respectively. The ECAR value after glycolysis inhibition with 2DG is subtracted from both the basal and maximal rates to discount non-glycolytic ECAR.

### Statistical analysis

Statistical analyses were performed using a two tailed Student’s t-Test, one-way or two-way Analysis of Variance (ANOVA) with Tukey’s correction for multiple comparisons. All results are presented as mean ± Standard Deviation or Standard Error. P values ≤ 0.05 were considered significant.

### Data availability

The DNA sequence and microarray data generated in this study will be deposited in public databases upon publication.

## Results

### The SELECT supplements decrease proliferation and induce cell death in LNCaP cancer organoids

**Figure 1:**
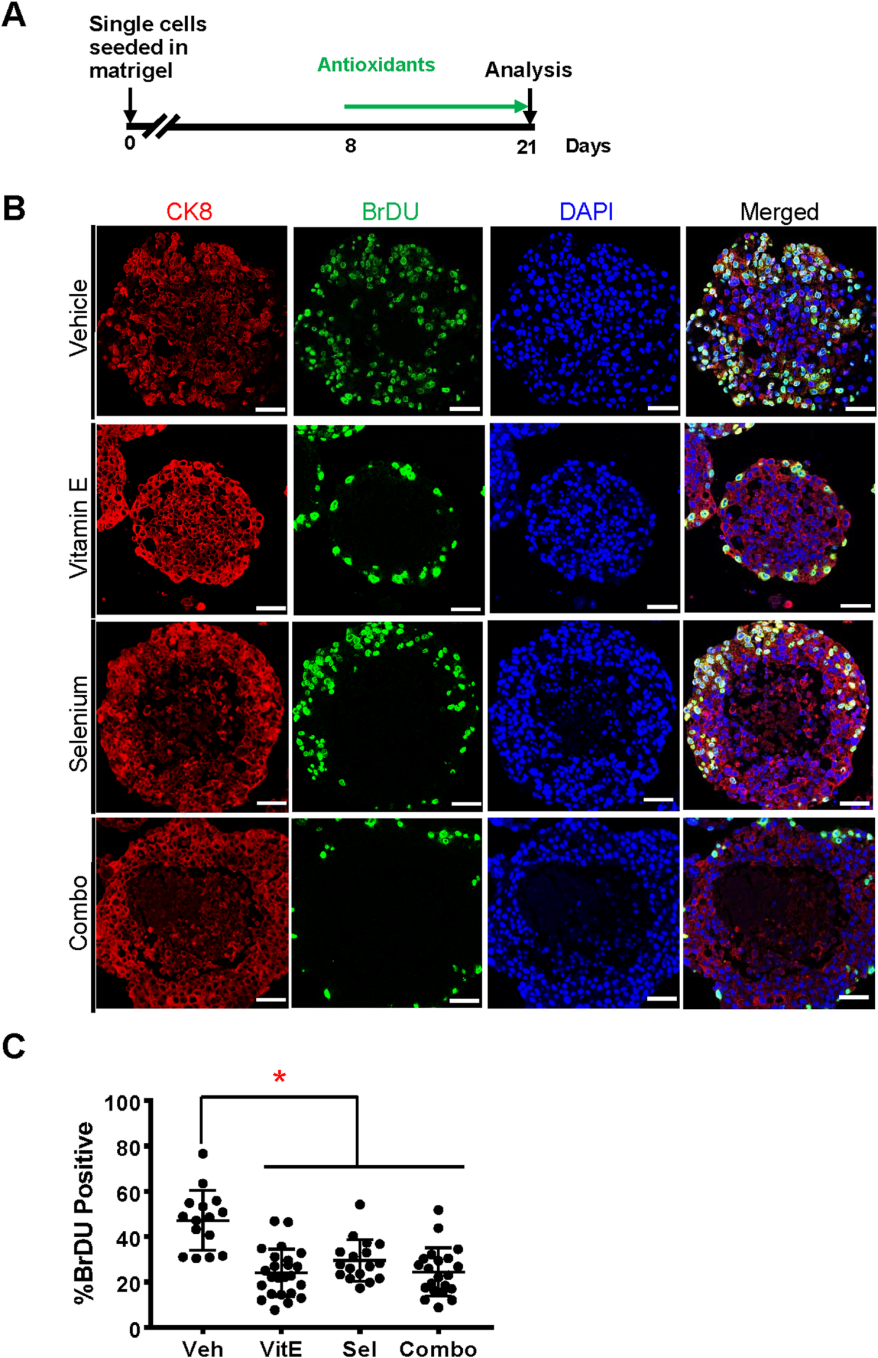
The SELECT antioxidants decrease proliferation and increase cell death in LNCaP organoids. LNCaP cells were grown in organoid media, treated with antioxidants then immunostained with anti-CK8, anti-BrdU antibodies and a DAPI counterstain. (**A**) Time line of the culture and treatment of organoids (**B**) LNCaP cells gave rise to luminal organoids displaying a significant decrease in proliferation and increased cell death when treated with antioxidants. (**C**) Quantification of BrDU incorporating cells as a percentage of total cells shows that the antioxidants significantly reduced the number of actively dividing cells. Scale bars represent 50 μm. Asterisks represent statistical significance (One way ANOVA with Tukey’s correction for multiple comparisons). *p ≤ 0.0001; error bars represent SD.

Laboratory studies showing anti-tumorigenicity of Vitamin E or Selenium in the prostate mostly utilized LNCaP, PC-3, and DU145 tumor cells. However the failure of SELECT led us to hypothesize that the response to antioxidants may depend on disease stage. To test this, we first evaluated the effects of the SELECT agents on LNCaP prostate cancer cell organoids (Fig. 1A). The human prostate epithelium contains basal, luminal and neuroendocrine cells distinguishable by the expression of specific markers^25^. LNCaP yielded purely luminal organoids (Fig. 1B). In agreement with previous reports^26^, the antioxidants strongly decreased BrDU incorporation relative to the vehicle control (Fig. 1B and C). Furthermore, while the cycling cells in the vehicle were dispersed throughout the organoids, those in the antioxidant treated organoids especially vitamin E were restricted to the outermost layer (Fig. 1B). Additionally, the antioxidants induced cell death especially in the inner, extra-cellular matrix (ECM) deprived cells (Fig. 1B) consistent with previous reports^27^.

### The SELECT supplements do not increase cell proliferation in benign primary prostate organoids

**Figure 2:**
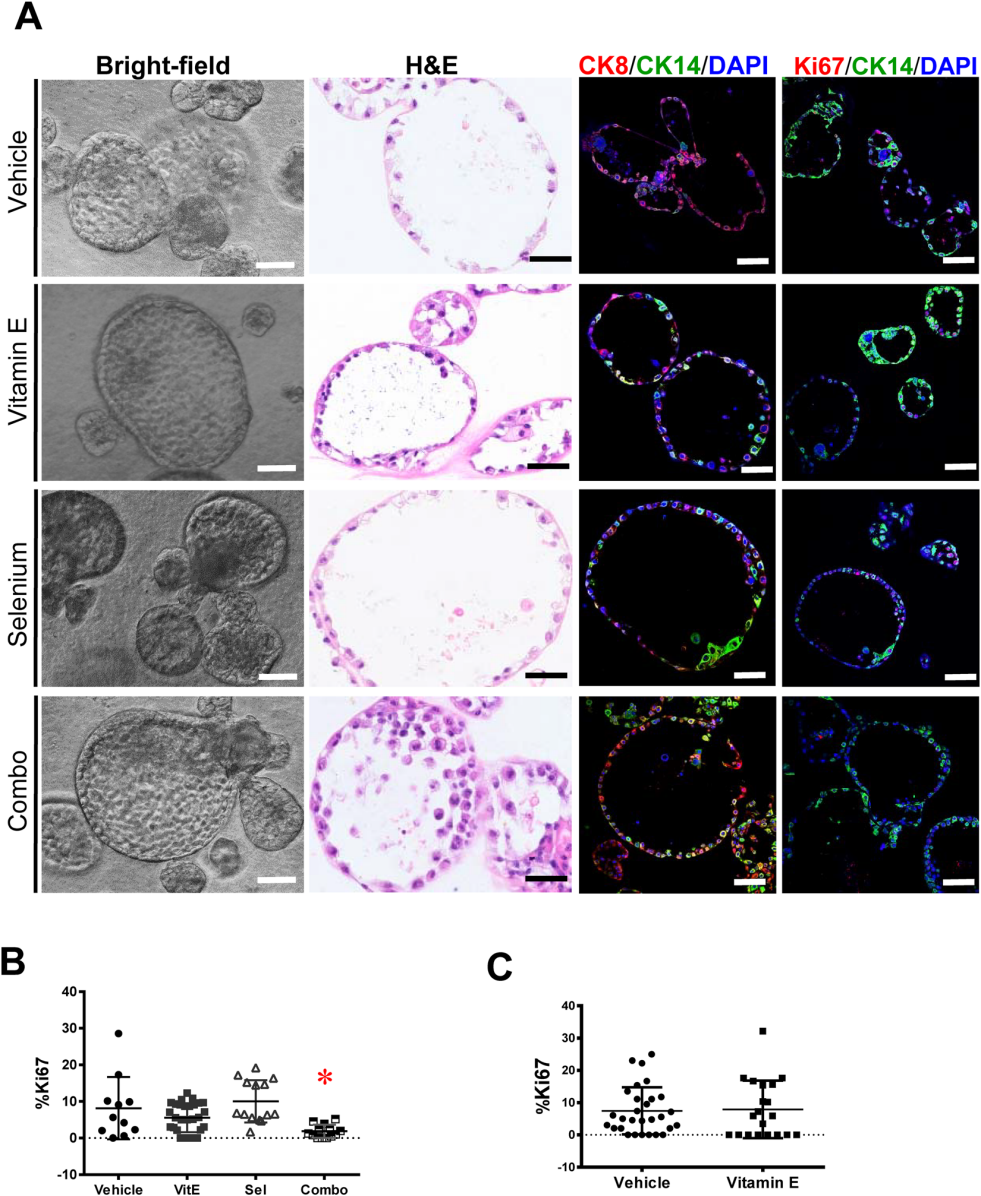
Vitamin E has no significant effect on the proliferation of healthy primary organoids. To model the SELECT trial, organoids from normal primary prostate cells of two subjects were treated with the SELECT agents. (**A**) Bright-field view showing several phenotypes of organoids from one of the subjects. The H&E stain shows the formation of large organoids with hollow lumens while the immuno-staining shows that these organoids expressed both luminal CK8 and basal CK14 epithelial markers. (**B**) Ki67 quantification showed no significant impact for Vitamin E or Selenium but their combination decreased organoid proliferation in the first subject. (**C**) Quantification of Ki67 in organoids from the second subject showed no significant difference in proliferation between vehicle and vitamin E treatments. Each data point represents a single field of view. Scale bars represent 100 μm for Brightfield and IF images and 50 μm H&E. Asterisks represent statistical significance (One way ANOVA with Tukey’s correction for multiple comparisons or a two tailed t-test). *p ≤ 0.05; error bars represent SD.

Next we tested the effects of the supplements on benign primary human prostate epithelial cell organoids. We generated organoids using primary prostate epithelial cells from two African American subjects without cancer as previously reported^16^. The absence of prostate cancer related alterations after the targeted sequencing of 222 cancer genes cells confirmed the cells to be benign (Supplementary Table 1). The primary organoids were heterogeneous in size, morphology and expression of basal or luminal markers (Fig. 2A). Notably however, antioxidant monotherapies did not affect the proliferation of these organoids while the combination treatment decreased Ki67 staining in the first subject (Fig. 2B and C).

### Vitamin E significantly increases cell proliferation in premalignant RWPE-1 prostate organoids but not in 2D culture

**Figure 3:**
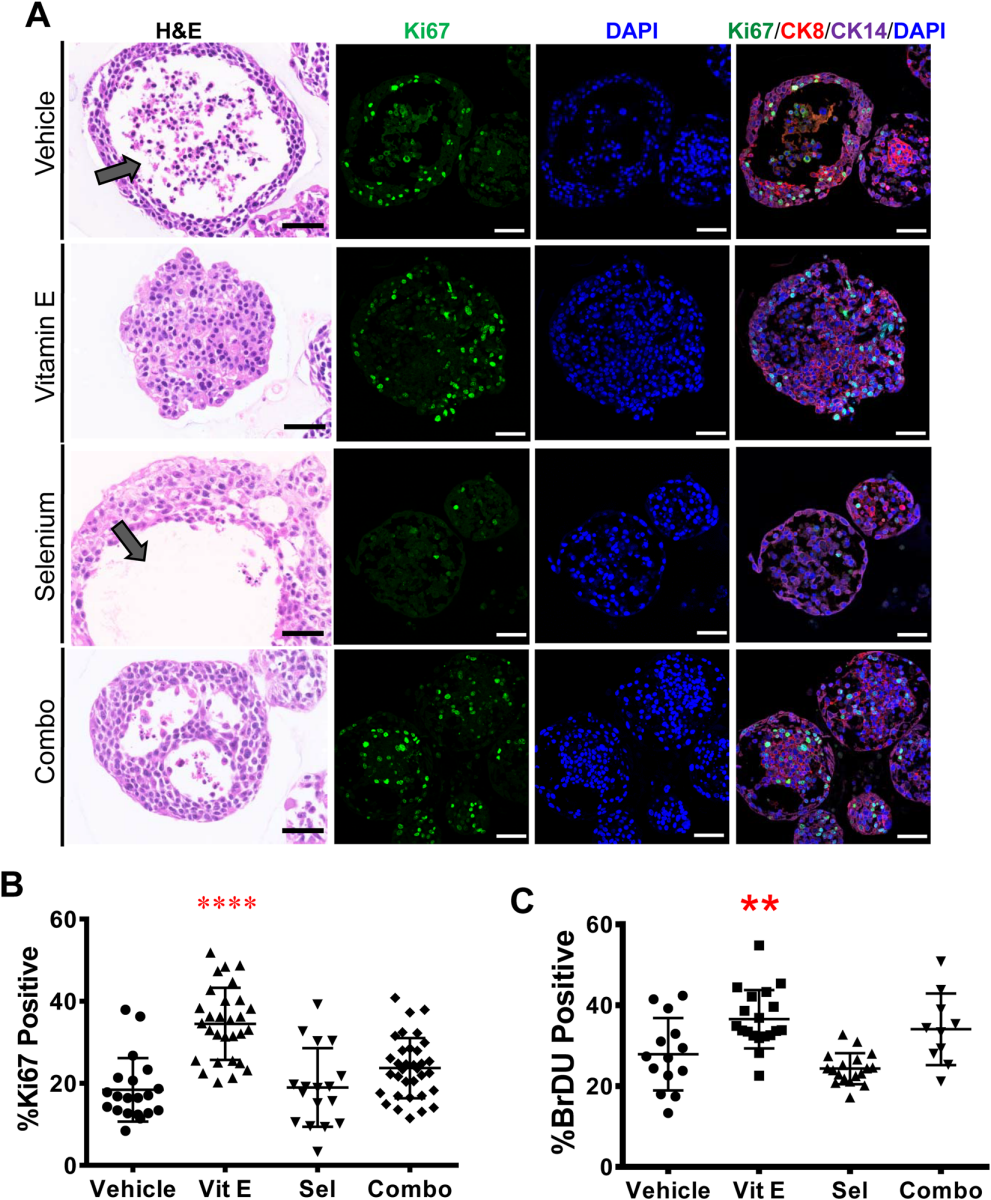
Vitamin E alone drives proliferation in organoids from the non-tumorigenic prostate cell line, RWPE-1, recapitulating the SELECT trial. Antioxidant-treated RWPE-1 organoids were sectioned and stained with H&E or anti-CK8, CK14 and Ki67 antibodies (**A**) H&E staining showed that organoids treated with Vehicle or Selenium had mostly hollow lumens (arrows) compared to the filled morphology in Vitamin E treated organoids. The confocal images show basal and luminal staining and increased Ki67 detection in Vitamin E treated organoids. (**B**) Quantification of the percentage of Ki-67 positive cells showed a highly significant increase in proliferation in organoids treated with Vitamin E alone. (**C**) Quantification of the percentage of BrDU incorporating cells in RWPE-1 organoids after two weeks of culture showed that Vitamin E alone significantly increased the number of dividing cells. Each data point represents a single field of view. Scale bars represent 50 μm. Asterisks represent statistical significance (One way ANOVA with Tukey’s correction for multiple comparisons). **p ≤ 0.005, ****p ≤ 0.0001; error bars represent SD.

The SELECT trial revealed a deleterious effect of Vitamin E on a fraction of individuals without prior evidence of prostate cancer^5^. Therefore we reasoned that these individuals might have harbored “initiated” cells in a pre-malignant state that were then pushed to malignancy by chronic antioxidant treatment.  We tested this on organoids generated from the immortalized but non-tumorigenic RWPE-1 human prostate epithelial cell line. RWPE-1 cells are immortalized with the E7 oncoprotein from HPV18^28^ which modulates the activity of the retinoblastoma (Rb) tumor suppressor^29^. While most RWPE-1 organoids treated with the vehicle or Selenium had hollow lumens, those treated with Vitamin E or the combination displayed marked luminal filling (Fig. 3A). Vitamin E caused a near two fold increase in proliferation with 35% Ki67 positivity compared to the vehicle at 19% (Fig. 3B). In contrast, Selenium had no impact on proliferation while the combination treatment had an intermediate effect at 18% and 25% Ki67 positivity respectively (Fig. 3B). Confirming these results, Vitamin E had the highest number of BrDU incorporating cells at 37%, however, Selenium (24%) and not the vehicle (27%) had the lowest (Fig. 3C). These *in vitro* RWPE-1 organoid findings where Vitamin E enhances proliferation while Selenium counteracts Vitamin E, are highly reminiscent of the clinical trial data from SELECT. In contrast, Vitamin E had no significant effect on the growth rate of RWPE-1 cells grown in 2D (Supplementary Fig. S1). In this condition, the combination of Vitamin E and Selenium significantly increased cell growth (Supplementary Fig. S1) indicating that 2D culture could not recapitulate the SELECT results.

### Proliferation in the premalignant organoids is independent of antioxidant structure or mechanism of action

**Figure 4:**
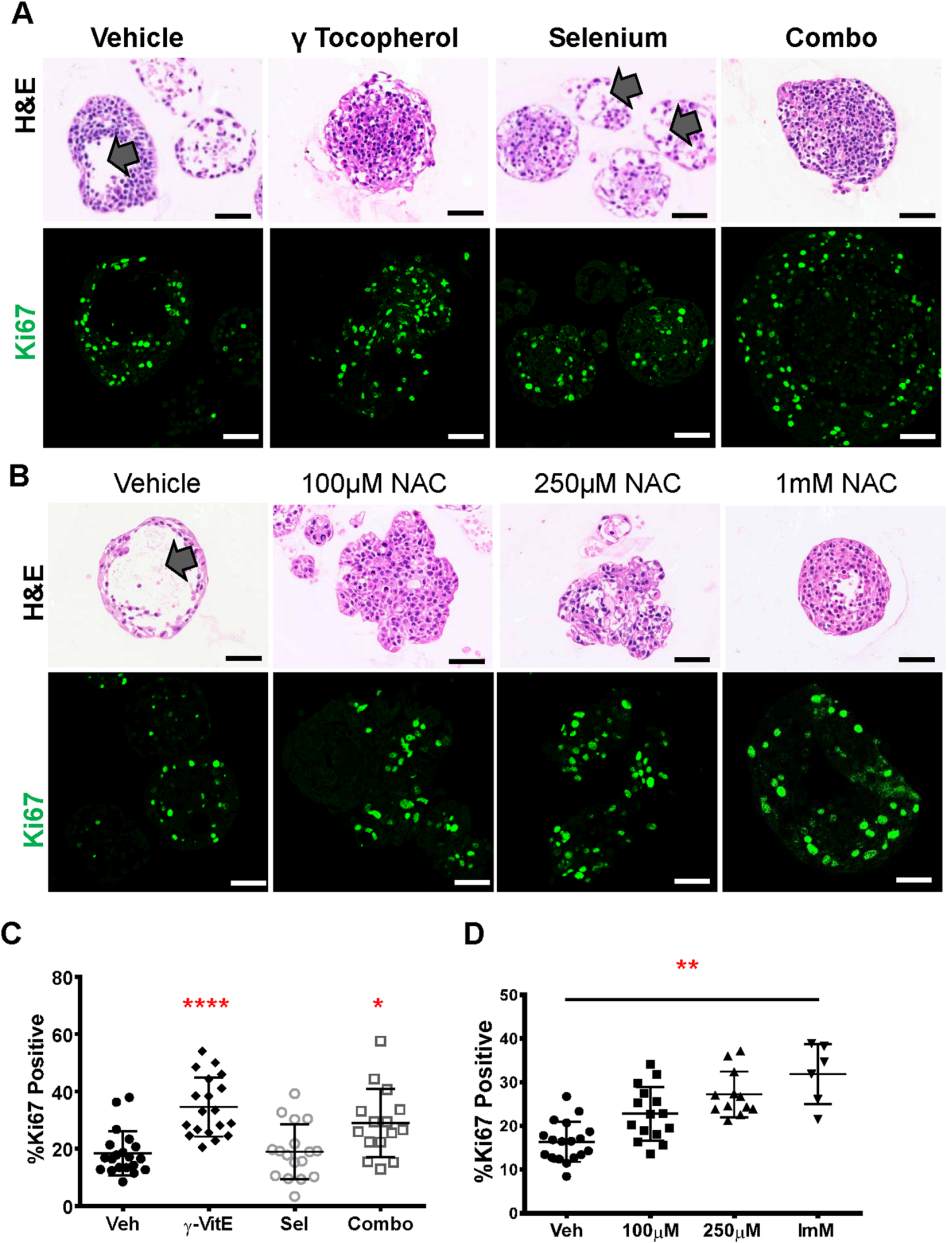
The proliferative phenotype in RWPE-1 organoids is independent of the Vitamin E isomer or antioxidant structure. (**A**) H&E staining showing hollow lumens (arrows) in vehicle and Selenium and filled lumens for γ-Tocopherol or its combination with Selenium. The confocal images show Ki67 immunostaining on sections of RWPE-1 organoids treated with the vehicle, γ-Tocopherol, its combination with Selenium or Selenium alone (**B**) H&E images showing hollow and filled morphology in vehicle and NAC treated organoids respectively and Ki67 immunostaining on sections of RWPE-1 organoids treated with vehicle or increasing concentrations of NAC. (**C**) Quantification of Ki-67 positive cells from (**A**) showed a significant increase in the proliferation of organoids treated with γ-Tocopherol or its combination with Selenium but not Selenium alone. (**D**) Quantification of Ki-67 positive cells from (**B**) showed a dose dependent increase in proliferation in organoids treated with NAC. Each data point represents a single field of view. Scale bars represent 50 μm. Asterisks represent statistical significance (One way ANOVA with Tukey’s correction for multiple comparisons). *p ≤ 0.05, **p ≤ 0.01, ****p ≤ 0.0001; error bars represent SD.

**Figure 5:**
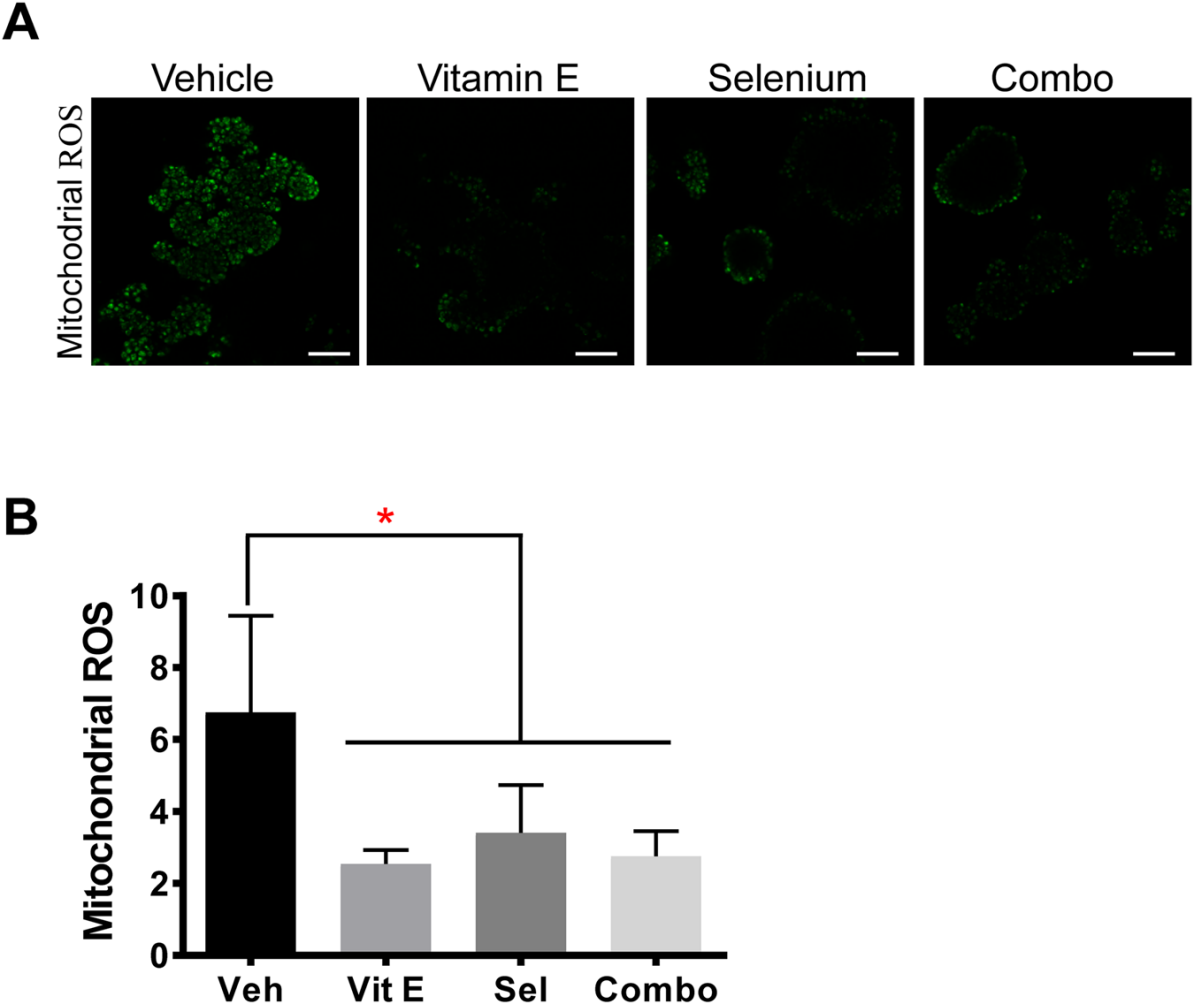
All SELECT agents significantly decreased mitochondrial ROS. To detect mitochondrial ROS in RWPE-1 organoids, we used a redox probe (CellROX Green) that is non-fluorescent in its reduced state but which fluoresces proportionately upon oxidation. (**A**) RWPE-1 organoids treated with the SELECT agents were stained with the probe and imaged by confocal microscopy. (**B**) Quantification of the mean fluorescence intensity of the images in (**A**) showed a significant reduction in mitochondrial ROS by all the SELECT agents. Scale bars represent 100 μm. Asterisks represent statistical significance (One way ANOVA with Tukey’s correction for multiple comparisons). *p ≤ 0.0001, error bars represent SD.

To test the effect of other antioxidants, we treated RWPE-1 organoids with a different Vitamin E isomer, γ-Tocopherol or NAC (Fig. 4). Organoids treated with γ-Tocopherol alone or in combination with Selenium had a filled lumen morphology (Fig. 4A). Further, γ-Tocopherol increased proliferation to 34% Ki67 positivity compared to vehicle at 19% (Fig. 4C). The combination of γ-Tocopherol and Selenium also had a higher proliferation rate at 29% (Fig. 4C). Therefore unlike α-Tocopherol (Fig. 3B), the addition of Selenium did not greatly attenuate the effect of γ-Tocopherol (Fig. 4C). While Vitamin E isomers are lipophilic antioxidants that prevent lipid peroxidation^30^, NAC is a precursor in the synthesis of the intracellular antioxidant glutathione^31^. NAC treated organoids had a dose dependent proliferation increase and filled lumens (Fig. 4B and D). Further, to determine whether the SELECT supplements affect the levels of ROS in RWPE-1 organoids, we quantified fluorescence in treated organoids stained with mitochondrial CellROX probes. The SELECT supplements significantly lowered mitochondrial ROS (Fig. 5A and B).

### Microarray analysis revealed opposing effects of Vitamin E and Selenium on cell proliferation in the premalignant organoids

**Figure 6:**
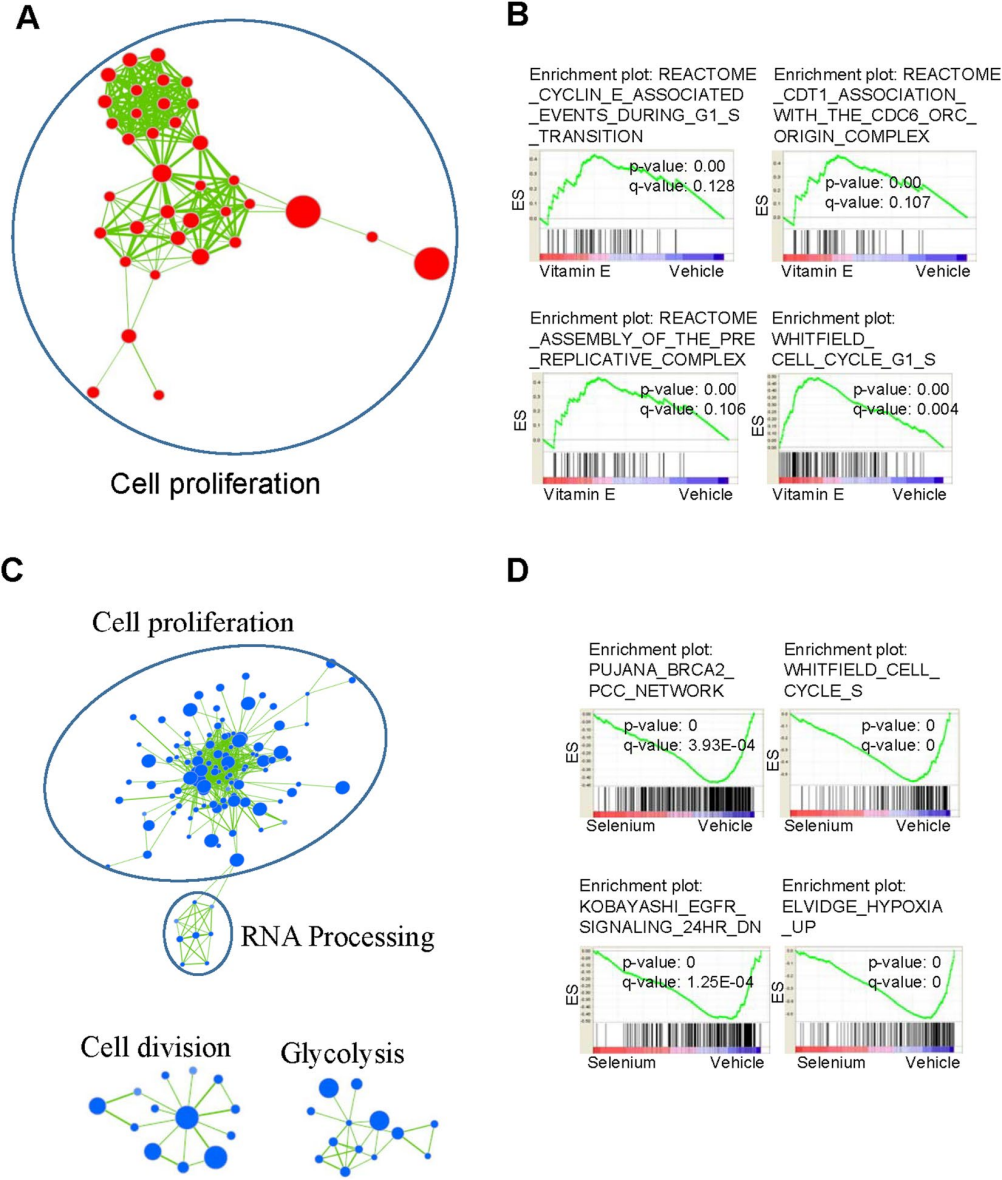
In RWPE-1 organoids, Vitamin E upregulates a cell proliferation gene network which is suppressed by Selenium. We used Cytoscape to cluster together functionally related gene sets found to be significantly enriched after querying our microarray data with MSigDB’s C2 curated gene sets using GSEA. We show networks containing ≥5 gene sets (False Discovery Rate q value < 0.25). Node size corresponds to gene set size. Red circles represent up-regulation and blue circles represent down-regulation of the gene set. Colour intensity represents significance by enrichment p value. Line thickness connecting the gene set nodes represents the degree of gene overlap between the two sets. (**A**)Vitamin E upregulated a gene set network associated with cell proliferation (**B**) Example GSEA enrichment plots for selected gene sets from the Vitamin E network. The Enrichment Score (ES; y-axis) reflects the degree to which a gene set was upregulated (cumulative positive score) or down-regulated (cumulative negative score) in the treatment group. Each vertical line in the ‘bar code’ represents a single gene in a gene set. Hue designates the direction in which the genes are altered (red = enriched in Vitamin E, blue = depleted in Vitamin E). Nominal p value (statistical significance of the enrichment) and the FDR are indicated. (**C**) Selenium downregulated cell proliferation, glycosis gene networks among others (**D**) Example GSEA enrichment plots for selected gene sets from the Selenium affected networks.

To gain further insight into the effects of SELECT supplements on RWPE-1 organoids we performed gene expression profiling using microarrays followed by gene set enrichment analysis (GSEA)^22^. Vitamin E upregulated cancer and cell cycle related gene sets which in contrast were suppressed by Selenium and the combination treatments (Supplementary Figs S2–S4). The leading edge subsets describe the genes that contribute most to a gene set’s enrichment score and thus have the highest correlation with the phenotype of interest. The leading edge analysis showed an upregulation of cell cycle and genome replication genes including cyclins and mini-chromosome maintenance proteins (MCM) by Vitamin E but downregulated by Selenium and the combination treatments (Supplementary Table 2). The GSEA results were visualized with the Cytoscape Enrichment Map plug-in which groups significant gene sets into functional networks based on annotation similarity and gene overlap^23,24^. The key network affected by all the treatments was cell proliferation but while it was upregulated by Vitamin E (Fig. 6A and B), it was suppressed by Selenium (Fig. 6C and D) and the combination treatment (Supplementary Fig. S5A and B).

### Suppression of glucose uptake leads to a drop in ATP generation in detached premalignant cells

**Figure 7:**
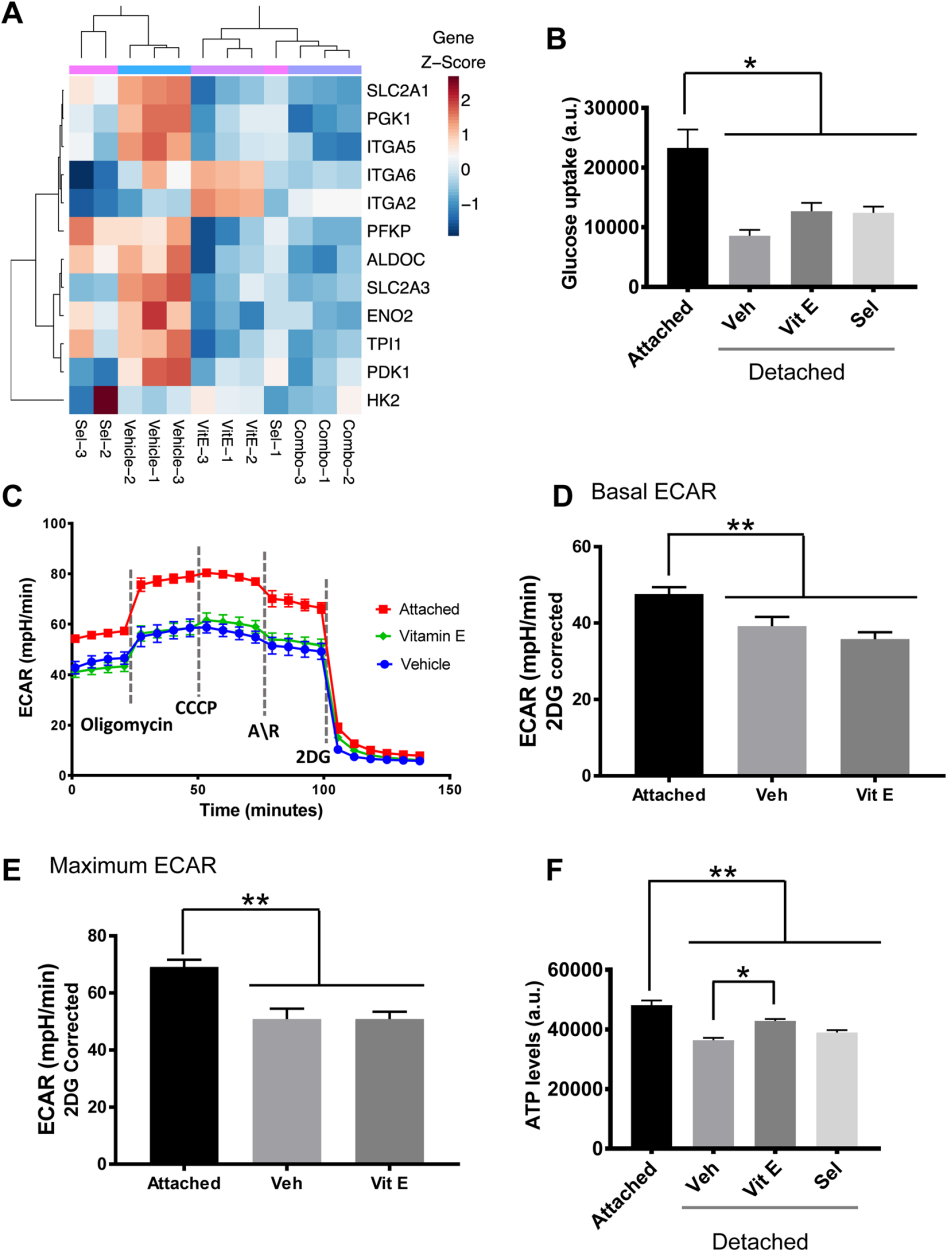
Vitamin E promotes cell survival through a metabolic rescue of ECM detached cells. (**A**) Heatmap of differentially expressed genes in the glycolysis and cell attachment pathways identified to be affected by the gene set analysis showed a downregulation of both pathways to varying degrees by antioxidant treatment (**B**) Measurement of glucose uptake in adherent or non-adherent, (poly-HEMA coated plates), RWPE-1 cells showed a significant decrease in the detached cells that was not rescued by the antioxidants after 24 h. (**C**) ECAR analysis of RWPE-1 cells grown in adherent and non-adherent conditions and treated with the SELECT supplements for 24 h, n = 15. (**D**) Basal ECAR was calculated by subtracting non-glycolytic ECAR from the basal ECAR measurements vs vehicle. (**E**) Maximal ECAR was calculated by subtracting non-glycolytic ECAR from the ECAR measurements after the addition of oligomycin. (**F**) ATP levels measured in 24 h RWPE-1 cell cultures in adherent or non-adherent plates showed a significant ATP rescue by Vitamin E treatment, (n ≥ 3). Asterisks represent statistical significance (One way ANOVA with Tukey’s correction for multiple comparisons). *p ≤ 0.001, **p ≤ 0.0001; error bars represent SD. A.U. means arbitrary units.

From the gene expression analysis, we observed a downregulation of glucose transporters and glycolytic enzymes to varying degrees among the treatments (Fig. 7A). Several integrins which mediate ECM cell attachment were also downregulated (Fig. 7A). Studies in mammary organoids have demonstrated differences in the glycolytic rates between the outer ECM attached and the inner detached cells^32^. We therefore tested whether suppression of the glycolysis pathway was associated with differences in glucose absorption and glycolysis. We used adherent and nonadherent cells to mimic the attached (outer) and detached (inner) organoid cells respectively. Cell detachment significantly reduced glucose absorption and this was not rescued by the addition of antioxidants (Fig. 7B). Consequently, detached cells had significantly lower levels of basal and maximal ECAR, a measure of lactate production from glycolysis, compared to attached cells which was not rescued by the addition of Vitamin E (Fig. 7C–E). Next we measured ATP levels under the same conditions to determine the effect of reduced glycolysis and antioxidants on cell energetics. The detached cells had significantly lower ATP levels compared to attached cells (Fig. 7F). Though Vitamin E did not rescue glycolysis, it did rescue the diminished ATP levels in detached cells (Fig. 7F) consistent with findings in mammary cells^32^.

### Vitamin E increases the survival of detached premalignant cells by stimulating fatty acid oxidation

**Figure 8:**
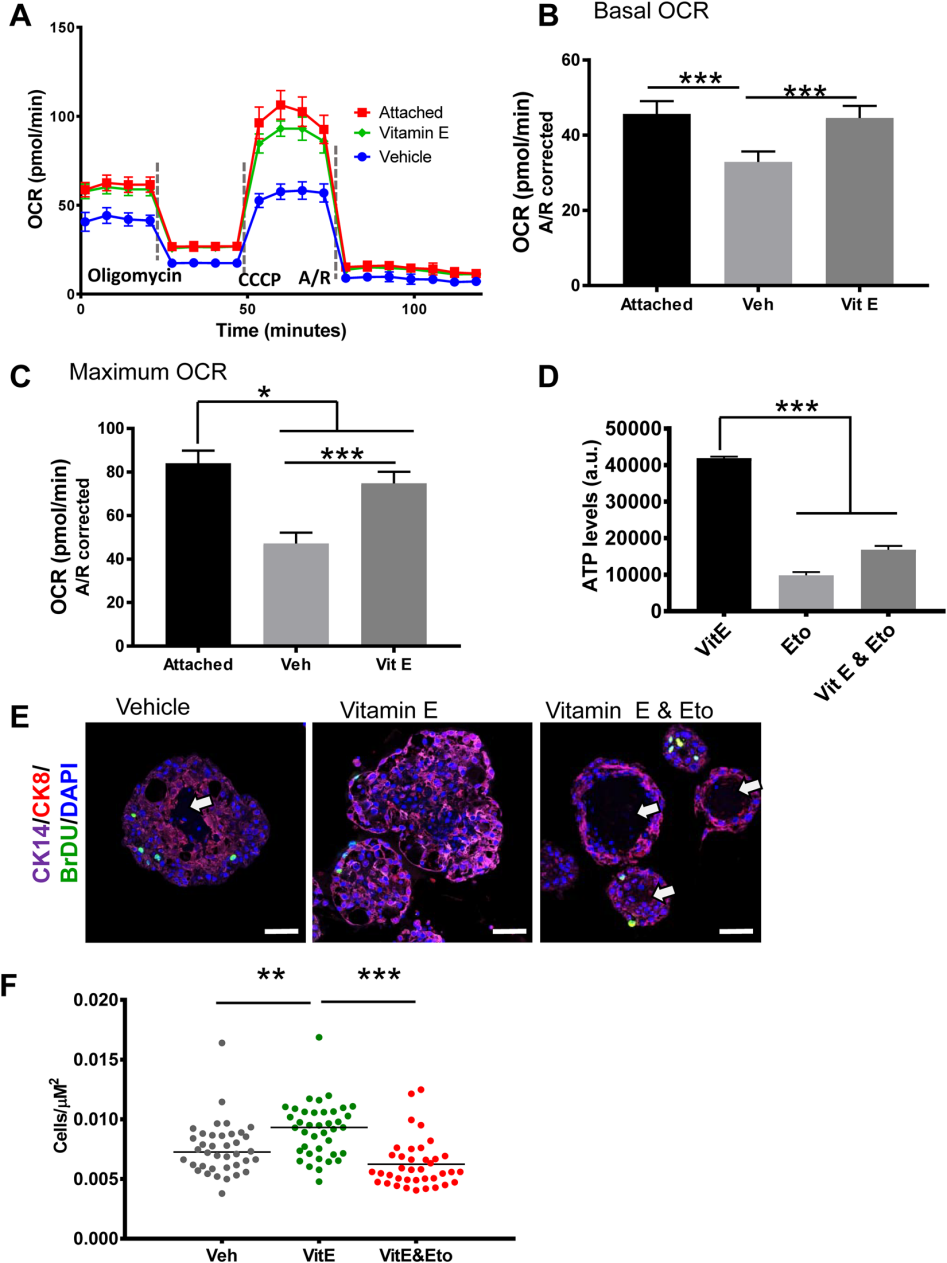
Vitamin E restores ATP levels in detached cells by stimulating fatty acid oxidation leading to organoid luminal filling. (**A**) OCAR analysis of RWPE-1 cells grown in adherent and non-adherent conditions and treated with the SELECT supplements for 24 h, n = 15. (**B**) Basal OCAR was calculated by subtracting non-mitochondrial OCAR from the basal OCAR measurements. (**C**) Maximal OCAR was calculated by subtracting non-mitochondrial OCAR from the OCAR measurements after the injection of CCCP. (**D**) Detached RWPE-1 cells were treated with vehicle, vitamin E or vitamin E and an FAO inhibitor, Etomoxir (Eto, 25 μM) for 24 h; ATP measurement showed that FAO inhibition abrogated the ATP rescue by Vitamin E. (**E**) Immunostained sections of RWPE-1 organoids from (**D**), showed that the Vitamin E treated organoids had filled lumens while those co-treated with Vitamin E and Etomoxir or vehicle had hollow lumens (arrows). (**F**) Cell density of organoids from (**E**) measured by dividing the number of total cells per organoid by its area showed that the Vitamin E organoids had the highest cell densities while those co-treated with Vitamin E and Etomoxir had the lowest cell densities. Scale bars represent 100 μm. Asterisks represent statistical significance (One way ANOVA with Tukey’s correction for multiple comparisons). **p ≤ 0.05, **p ≤ 0.01, ***p ≤ 0.0001, error bars represent SD.

Next we sought to determine whether this ATP rescue despite the decreased glycolytic flux was through increased oxidative phosphorylation, the other major pathway for energy generation in cells. To do this we measured changes in oxygen consumption rate (OCR) which is linked to mitochondrial oxidative phosphorylation. While vehicle treated detached cells had low basal and maximum OCR, those treated with Vitamin E had significantly higher OCR levels that were comparable to the attached cells (Fig. 8A–C). When glucose is depleted, cells can derive energy from fats through fatty acid oxidation (FAO). Since Vitamin E did not rescue glucose uptake, we tested whether it might stimulate FAO. ATP levels were measured in non-adhering, antioxidant treated RWPE-1 cells with or without Etomoxir, an FAO inhibitor. FAO inhibition abrogated the ATP rescue by antioxidants (Fig. 8D). To move these findings to a more physiologically relevant model, we tested the effect of FAO inhibition in Vitamin E-treated RWPE-1 organoids. Intriguingly, FAO inhibition in Vitamin E treated organoids selectively killed the inner, ECM detached cells, reverting the filled lumen morphology back to a normal hollow morphology (Fig. 8E). Consequently, Vitamin E treated organoids had the highest cell densities while those co-treated with etomoxir had the lowest cell densities pointing to increased cell survival (Fig. 8F).

## Discussion

The role of antioxidants in cancer chemoprevention has been controversial. The surprising findings from the SELECT trial showing an increased risk of prostate cancer with Vitamin E supplementation^5^ have been the subject of much discussion. Our key contribution to this debate is our finding that, premalignant but not benign or malignant prostate epithelial cells grown as organoids respond to antioxidant treatment in a manner that recapitulates the findings of the trial. Since cells at different stages of tumorigenesis experience different levels of ROS, it is reasonable to expect the antioxidant effect to be dependent on a cell’s position in the tumor evolution spectrum. The supplements decreased proliferation and increased cell death in the LNCaP cancer organoids, consistent with reports that show antioxidant efficacy in established cancer cell lines^26,27^. The LNCaP organoid data therefore supports the concept that moderate levels of ROS damage DNA leading to mutations that can aggravate cancer^33^.

The SELECT supplements however did not affect the proliferation of benign primary prostate epithelial cells. On the contrary, the agents with the exception of Selenium significantly increased the proliferation of ‘pre-initiated’ RWPE-1 cell organoids. These results are consistent with SELECT where only a fraction of the subjects on Vitamin E had an increase in PCa risk^5^. We suggest that these individuals might have harbored initiated, pre-malignant cells that were pushed in to malignancy by Vitamin E. Our group has previously shown that NAC promotes proliferation in the prostates of Nkx3.1^−/−^ mice, which develop premalignant prostatic epithelial hyperplasia, but not wild type mice^34^. Furthermore, a functional variant in the *NKX3.1* gene was associated with the increased risk of prostate cancer in subjects from the Selenium and Vitamin E Cancer Prevention Trial (SELECT)^35^. This points to the importance of the underlying genetic background of prostate cells in modifying the response to antioxidant supplementation.

The use of organoid culture allowed us to mimic the spatial constraints on solid tumor cells *in vivo*. As tumors expand, cells experience a nutrient and oxygen exposure gradient. Likewise, the central cells in large organoids are under various stresses including loss of ECM attachment and limits in the diffusion of nutrients and oxygen^15^. In the normal organoids, the cells at the center die off since nutrient uptake depends on the ECM attachment status and growth factor signaling^36^. Similarly, ECM detachment in the vehicle treated premalignant organoids reduced glucose uptake, glycolysis and ATP levels which diminished cell survival leading to hollow lumens. Vitamin E treatment on the other hand rescued the ATP deficiency in a fatty acid oxidation-dependent manner which increased premalignant cell survival leading to filled lumens, (summarized in Supplementary Fig. S6). In comparison, LNCaP cells in the control malignant organoids continued to proliferate even when detached. The accumulation of multiple alterations allows cancer cells to circumvent extracellular regulation enabling them to uptake nutrients constitutively^36^.

Finally in this study, just like in SELECT, the effects of Selenium were more complex. We found that in addition to decreasing ROS, Selenium had broader effects on gene expression in the RWPE-1 organoids compared to Vitamin E. Selenium functions through incorporation in to Selenoproteins with a broad range of activities besides redox homeostasis^37–39^. We hypothesize that any protumorigenic effects of Selenium’s antioxidant function are counteracted by its effect on other anti-tumorigenic pathways. However, although Selenium treatment induced an anti-proliferative gene signature, it did not lower the proliferation index in RWPE-1 organoids. This could be explained by the existence of alternative mechanisms that override transcriptional regulation.

This study was limited by the difficulty of establishing cultures of pre-malignant prostate epithelial cells *in vitro* so we used the RWPE-1 cell line instead. Additionally, we did not directly measure FAO but used the more general mitochondrial respiration readout, OCR. Future studies could examine the effect of antioxidant supplementation in the presence of early event mutations associated with PCa initiation like NKX3.1 downregulation or MYC overexpression^25^. The mechanism(s) through which Vitamin E increases cell proliferation in the premalignant state also remain undetermined. In summary, our data suggest that antioxidants could be effective against malignant PCa but they promote tumorigenesis in premalignant cells.

## Electronic supplementary material

Supplementary Information
