# Supplementary material for: Organoids model distinct Vitamin E effects at different stages of prostate cancer evolution
Source: Sci Rep. 2017 Nov 24;7:16285. doi: 10.1038/s41598-017-16459-2 (PMC5701195; doi:10.1038/s41598-017-16459-2)

SUPPLEMENTARY INFORMATION

Organoids model distinct Vitamin E effects at different stages of prostate cancer evolution

Rose N. Njoroge[1], Kenji Unno[1], Jonathan C. Zhao[2], Anum F. Naseem[1], Jonathan F. Anker[1], Warren A. McGee[3], Larisa Nonn[4], Sarki A. Abdulkadir[1,5 *]

Supplementary Tables:

Supplementary Table S1. Summary of non-synonymous mutations found from the targeted sequencing of 222 cancer genes in the primary cells from the two subjects used to generate benign organoids.

Subject 1								
Gene	Chrom	Exon	cDNA Change	Protein Change	Mutation Type	dbSNP142	OMIM Disease Association	ClinVar
HNF1A	chr12	9	c.A1720G	p.S574G	missense	rs1169305	Diabetes mellitus, insulin-dependent	Pathogenic
IL7R	chr5	4	c.G412A	p.V138I	missense	rs1494555	Severe combined immunodeficiency	Pathogenic
IL7R	chr5	2	c.T197C	p.I66T	missense	rs1494558	Severe combined immunodeficiency	Pathogenic
PRSS1	chr7	2	c.A86T	p.N29I	missense	rs111033566	Pancreatitis, hereditary	Pathogenic
APOE	chr19	4	c.T388C	p.C130R	missense	rs429358	Hyperlipoproteinemia, type III /Alzheimers	Pathogenic
ATM	chr11	20	c.T2927C	p.V976A	missense	rs146145357	Unavailable	Uncertain significance
LRRK2	chr12	1	c.G149A	p.R50H	missense	rs2256408	Parkinson disease 8	Uncertain significance
LRRK2	chr12	34	c.T4939A	p.S1647T	missense	rs11564148	Parkinson disease 8	Uncertain significance
LRRK2	chr12	49	c.T7190C	p.M2397T	missense	rs3761863	Unavailable	Uncertain significance
NSD1	chr5	5	c.G1811T	p.R604L	missense	rs61744451	Unavailable	Uncertain significance

Subject 2								
Gene	Chrom	Exon	cDNA Change	Protein Change	Mutation Type	dbSNP142	OMIM Disease Association	ClinVar
PRSS1	chr7	2	c.A161G	p.N54S	missense	rs144422014	Pancreatitis, hereditary	Pathogenic
PRSS1	chr7	2	c.C47T	p.A16V	missense	rs202003805	Pancreatitis, hereditary	Pathogenic
HNF1A	chr12	9	c.A1720G	p.S574G	missense	rs1169305	Diabetes mellitus, insulin-dependent, 20	Pathogenic
PRSS1	chr7	2	c.A86T	p.N29I	missense	rs111033566	Pancreatitis, hereditary	Pathogenic
LRRK2	chr12	1	c.G149A	p.R50H	missense	rs2256408	Unavailable	Uncertain significance

Supplementary Table S2. Selected leading edge genes from a sample of gene sets significantly enriched in the SELECT supplement treated premalignant organoids

Selected Leading Edge Genes Up-regulated by Vitamin E	
**Gene Set**	**Leading Edge Genes**
Reactome Cyclin E Associated Events During G1- S Transition	CCNH CCNE2 PSMA3 CCNA1 RB1 PSMA4 CDK7 PSME2 PSMA5 PSMB3 PSMD11 PSMD12 PSMA7 WEE1 MNAT1 RPS27A PSMA6 PSMC2 PSMA1 PSMD7 PSMA2PSMC6 PSMD14 PSMB9 SKP1 MAX PSMB7 PSMB5 PSMD6 PSMC5 PSMB1
Reactome CDT1 Association With CDC6 ORC Origin Complex	PSME4 CDC6 PSMA3 MCM8 PSMA4 ORC3 ORC5 PSME2 ORC4 PSMA5 PSMB3 PSMD11 PSMD12 PSMA7 RPS27A PSMA6 PSMC2 PSMA1 PSMD7 GMNN PSMA2 PSMC6 PSMD14 PSMB9 PSMA8 PSMB7 PSMB5 PSMD6 PSMC5 PSMB1 ORC6
Reactome Assembly Of The Pre Replicative Complex	PSME4 CDC6 PSMA3 MCM8 E2F3 PSMA4 ORC3 ORC5 PSME2 ORC4 PSMA5 MCM6 PSMB3 PSMD11 PSMD12 PSMA7 RPS27A PSMA6 PSMC2 PSMA1 PSMD7 GMNN PSMA2 PSMC6 PSMD14 PSMB9 PSMA8 PSMB7 PSMB5 PSMD6 PSMC5 PSMB1 ORC6
Whitfield Cell Cycle G1-S	SLC25A27 DNAJC3 SERPINB3 HELLS TTC14 ZRANB2 LUC7L3 ZNF141 NPAT CCNE2 FAM111B CDC6 LOC400879 RAB23 PCNAP1 PNN PMS1 ARGLU1 TIPIN SLC25A36 LNPEP NKTR ANKRD10 MDM1 INTS8 CLSPN DONSON CREBZF USP53 SEC62 SPIN3 DTL CEP57 NUP43 ACYP1 RNPC3 TOPBP1 C14ORF142 ATAD2 OSBPL6 MSH2 NASP TRA2A NEAT1 MCM6 ZNF852 FBXL20 PCNA DNAJB9 DIS3 CAPN7 WDR76 IVNS1ABP BRD7 SPIN4 CASP8AP2 MBOAT1 RNF113A SSR3 GINS3 TAF15 EIF2A GMNN
**Selected Leading Edge Genes Down-regulated by Selenium**	
**Gene Set**	**Leading Edge Genes**
Whitfield Cell Cycle S	CRLS1 RAD18 KIAA1598 RMI1 UBL3 KDELC1 CALM2 SVIP MASTL NRD1 LIPH NAB1 MBD4 GPR126 NT5DC1 NFE2L2 CDC45 EIF4EBP2 TRIM45 PILRB DYNC1LI2 ZNF217 ABHD10 PHTF2 DNAJB4 CASP2 PHOSPHO2 PRIM1 MAP3K2 MAN1A2 DCAF16 BMI1 RRM2 RAD51 EXO1 ABCC5 BRCA1 CALD1 MYCBP2 DONSON FAM178A ZWINT SLC22A3 TYMS USP1 BBS2 ENOSF1 DHFR NUP160 TMCC1 EFHC1 PHTF1 INTS7 HIST1H4H ESCO2 NSUN3 STAG3L1 SRSF5 KAT2B MCM8 FANCI PHIP ANKRD18A INSIG2 CDC7 PTAR1 ATAD2 ZBED5 SLC38A2 SRSF10 DMXL2 BRIP1 NEAT1 OGT C5orf42 SLC25A27 POLA1 LYRM7 TOP2A HELLS BIVM CREBZF DNA2 CCDC14 CCDC84 GOLGA8A GOLGA8B LOC389831 ANKRD36 CHML
Elvidge Hypoxia Up	AHNAK2 DTNA CYP1B1 CSRP2 BBX ATXN1 ALDOC IGFBP3 DPYSL2 GYS1 MET BNIP3 ANKZF1 YEATS2 MXI1 NDRG1 SORL1 GBE1 JUN DSC2 FAM13A SAMD4A SLC2A1 SRD5A3 TNFAIP8 CD59 KLF7 TRA2A EGFR SFXN3 CAV1 PGK1 FAM162A SPOCK1 TMEFF1 BNIP3L SLCO4A1 INSIG2 RLF ANG CCNG2 KRT7 EGLN1 VEGFC ENO2 DAAM1 VLDLR TXNIP GJA1 HK2 KDM3A PDK1 ZMYND8 DST TMEM45A SRPX LOXL2 RBPJ ANGPTL4 PAM TGFBI ERO1L P4HA1 ZNF292 WSB1 LOX PGAP1 ITPR1 EGLN3 CA9 STC1
Dacosta UV Response via ERCC3 TTD Down	UBXN7 DNAJC2 AVL9 MSH3 ACAP2 ITCH INTS3 ARHGEF10 GPATCH8 NFIB SLC16A7 ATXN1 SKAP2 RB1CC1 ROR1 AMPH NAV3 WDR37 SON PIK3C2A TMCC1 ARAP2 SERPINB2 FAM179B KLHL20 KIAA0922 PLCE1 MPHOSPH9 VPS13B USP15 TEAD1 PHF14 PDS5B CDC42BPA PVRL3 WDHD1 MALT1 TSC22D2 DOCK9 DOCK4 DST HERC4 BTAF1 LRP6 BICD1 HAS2
Kobayashi EGFR Signaling 24HR Down	KIF2C NCAPD3 ABCE1 KIF4A CDK1 PRIM1 GPSM2 RACGAP1 POLA2 GINS1 RRM2 NAA15 CDK2 NUDT15 RAD51 EXO1 MET RAD54B DUSP6 BRCA1 VRK1 DONSON ZWINT ZC3HAV1 NEK2 TYMS USP1 MCM6 SHCBP1 NCAPG2 MAD2L1 DHFR GJB3 PRC1 KIF15 BUB1 DNAJC9 DSN1 DKK1 TPX2 CCNA2 ITGA6 DCBLD2 ECT2 STEAP1 MELK TFPI2 TMEM194A SLCO4A1 FAM111A ELL2 MKI67 DEPDC1 FANCI CEP55 NT5E STIL SPC25 SMC4 ATAD2 NOC3L KIF23 MYBL1 PNN ERCC6L ENO2 KIF14 ZWILCH DTL DUSP4 NUSAP1 NRG1 PLK4 DLGAP5 LMNB1 CCNE2 SRSF7 PBK POLA1 NDC80 NCAPG HMMR TOP2A HELLS SMC2 HMGA2 ASPM STC1
Pujana BRCA Centered Network	NFYB RAD54L MCM4 DCP2 TTF2 RBBP8 SNRPA1 UBE2C RRM1 RFC3 POLR2B RAD21 SMC1A PAICS RPIA UNG DEK PPP2R5C PRKDC HMGB2 POLE RBBP4 NAE1 LBR BUB3 PPP1CC PCNA ZNF330 AURKA MSH2 NCK1 CDK1 PSIP1 GINS1 H2AFV RB1 SUZ12 BRCA1 SSBP2 NASP SMC3 MCM6 TOPBP1 MAD2L1 DHFR XPO1 MED20 TCERG1 MAT2A CCNA2 BRCA2 TMEM194A FANCI SMC4 CDC7 MRE11A RECQL LMNB1 SKP2 DDX46 ATM SRSF11 POLA1 NDC80 HMMR TOP2A SMC2 DNA2
**Selected Leading Edge Genes Down-regulated by Combination Treatment**	
**Gene Set**	**Leading Edge Genes**
Rosty Cervical Cancer Proliferation Cluster	TACC3 DNA2 MAD2L1 ACACA KIF15 PAQR4 ATAD2 CHEK1 HELLS CA2 CDC20 MELK AURKB E2F8 EBP CENPA CCNE2 TRIP13 PCNA AURKA FANCI KIFC1 H2AFX ERCC6L TTK PTTG1 CDCA3 TK1 SHCBP1 RAD51AP1 STIL TYMS POLA2 UBE2C KIF20B KIAA0101 FOXM1 RACGAP1 GINS1 RRM2 MYBL2 ZWINT PLK1 CDCA8 HJURP KIF4A DTL NCAPH NCAPG CENPF NUSAP1 CCNB2 KIF23 CCDC109B CDK1 TOP2A LMNB1 NEK2 PRC1 BUB1 KIF14 CCNA2 CENPE POLQ HMMR TPX2 PBK CEP55 MKI67 CCNB1 KIF20A TMPO KIF2C NDC80 DLGAP5 ASPM
Elvidge Hypoxia by DMOG Up	DTNA VLDLR S100A4 SCNN1B MXI1 AHNAK2 DSC2 SPRY1 PLAUR PFKP SRD5A3 BNIP3L KLF6 NDRG1 EGFR EGLN1 CITED2 KLF7 FAM13A IGFBP3 ANG BNIP3 CAV1 KDM3A TMEM45A SFXN3 SAMD4A P4HA1 SOX9 PDK1 VEGFC FAM162A GYS1 ERO1L TXNIP SLCO4A1 PGK1 SLC2A1 ASPH JUN ALDOC PAM HK2 GJA1 TGFBI SRPX ENO2 ANGPTL4 LOX LOXL2 ITPR1 EGR1 EGLN3 CA9 STC1
Chang Cycling Genes	PWP1 BARD1 KDM5B MCM5 FAM72B SDC1 CASP3 HN1 DHFR ARL4A ANP32E CCNF ASF1B WDR76 SCML1 WSB1 GINS3 TACC3 TUBA4A CKAP2 MAD2L1 ATAD2 HELLS NCAPD2 UHRF1 MELK TUBB4B CENPA CDKN3 NEAT1 TRIP13 PCNA DEPDC1 AURKA MCM6 KIF22 KIFC1 H2AFX ESCO2 PTTG1 RAD51AP1 FAM83D DEPDC1B UBE2C KIF20B KIAA0101 IFIT1 FOXM1 CDCA7 RRM2 CDC25C PLK1 DIAPH3 CDCA8 HJURP NCAPH CENPF NUSAP1 CCNB2 KIF23 CDK1 TOP2A LMNB1 BUB1 SKA3 CCNA2 CKAP2L GAS2L3 HMMR TPX2 PBK NUF2 FAM111B DLGAP5 PRR11 ANLN
Chiang Liver Cancer Subclass Proliferation Up	PLBD1 OIP5 PM20D2 WSB1 MMP9 CDCA7L TUBA4A ETV4 SPHK1 MAD2L1 MEP1A CDC7 HELLS CDC20 AURKB FUNDC1 E2F8 NCEH1 CDKN3 TRIP13 SKA1 DEPDC1 ARHGAP18 AURKA TMEM51 FANCI HIST1H4C ORC6 SOX4 TTK LAMB1 PTTG1 BACE2 SHCBP1 RAD51AP1 MARCKSL1 MMP12 DEPDC1B UBE2C SLC39A10 FOXM1 RACGAP1 UGCG CDCA7 ZWINT PELI1 HJURP TRNP1 KIF4A DTL NCAPG CENPF SOX9 NUSAP1 CCNB2 KIF23 CCDC109B CDK1 TOP2A LMNB1 NEK2 PRC1 KIF14 CCNA2 CKAP2L CENPE TPX2 PBK CEP55 MKI67 NUF2 CCNB1 KIF20A HK2 KIF2C PAG1 NDC80 DLGAP5 PRR11 ANLN ASPM

Supplementary Figure Legends:

Supplementary Figure S1. RWPE-1 cells in 2D do not recapitulate the results of the SELECT trial. Percent confluence over time of RWPE-1 cells with SELECT supplement treatment. Asterisks represent statistical significance (Two way ANOVA with Tukey's correction for multiple comparisons). *$p \leq 0.05$, ****$p \leq 0.0001$; error bars represent SD (n = 6 – 9).

Supplementary Figure S2. The combination of Vitamin E and Selenium suppresses cell proliferation, glycolysis and lipid metabolism pathways in RWPE-1 organoids. (A) Cytoscape visualization of gene sets enriched in RWPE-1 organoids treated with a combination of Vitamin E and Selenium. We show network with ≥ 5 functionally related gene sets (False Discovery Rate q value < 0.25). (B) Example GSEA enrichment plots for selected gene sets making up the networks shown in (A). Nominal p value (statistical significance of the enrichment) and the FDR are indicated.

Supplementary Figure S3. Top 30 gene sets that are significantly enriched by Vitamin E identified by gene set enrichment analysis (GSEA) of differentially expressed genes (p value ≤ 0.005)

Supplementary Figure S4. Top 30 gene sets that are significantly enriched by Selenium identified by gene set enrichment analysis (GSEA) of differentially expressed genes (p value ≤ 0.005).

Supplementary Figure S5. Top 30 gene sets that are significantly enriched by Vitamin E and Selenium combined identified by gene set enrichment analysis (GSEA) of differentially expressed genes (p value ≤ 0.005).

Supplementary Figure S6. Model of the impact of antioxidants on ATP generation depending on ECM attachment status. Attached cells have normal glucose uptake, high ATP and low ROS levels from glucose

oxidative phosphorylation and PPP flux which generates NADPH a reducing equivalent. When cells are detached however, their glucose uptake decreases due to a decrease in glucose transporter expression diminishing ATP generation. The PPP flux also decreases leading to ROS accumulation which further suppresses ATP levels by inhibiting fatty acid oxidation (FAO). Endogenous antioxidant supplementation neutralizes ROS which activates FAO restoring ATP levels and increases cell survival. FAO inhibition with Etomoxir abrogates the antioxidant ATP rescue diminishing cell survival in antioxidant treated detached cells.

Supplementary Figure S1

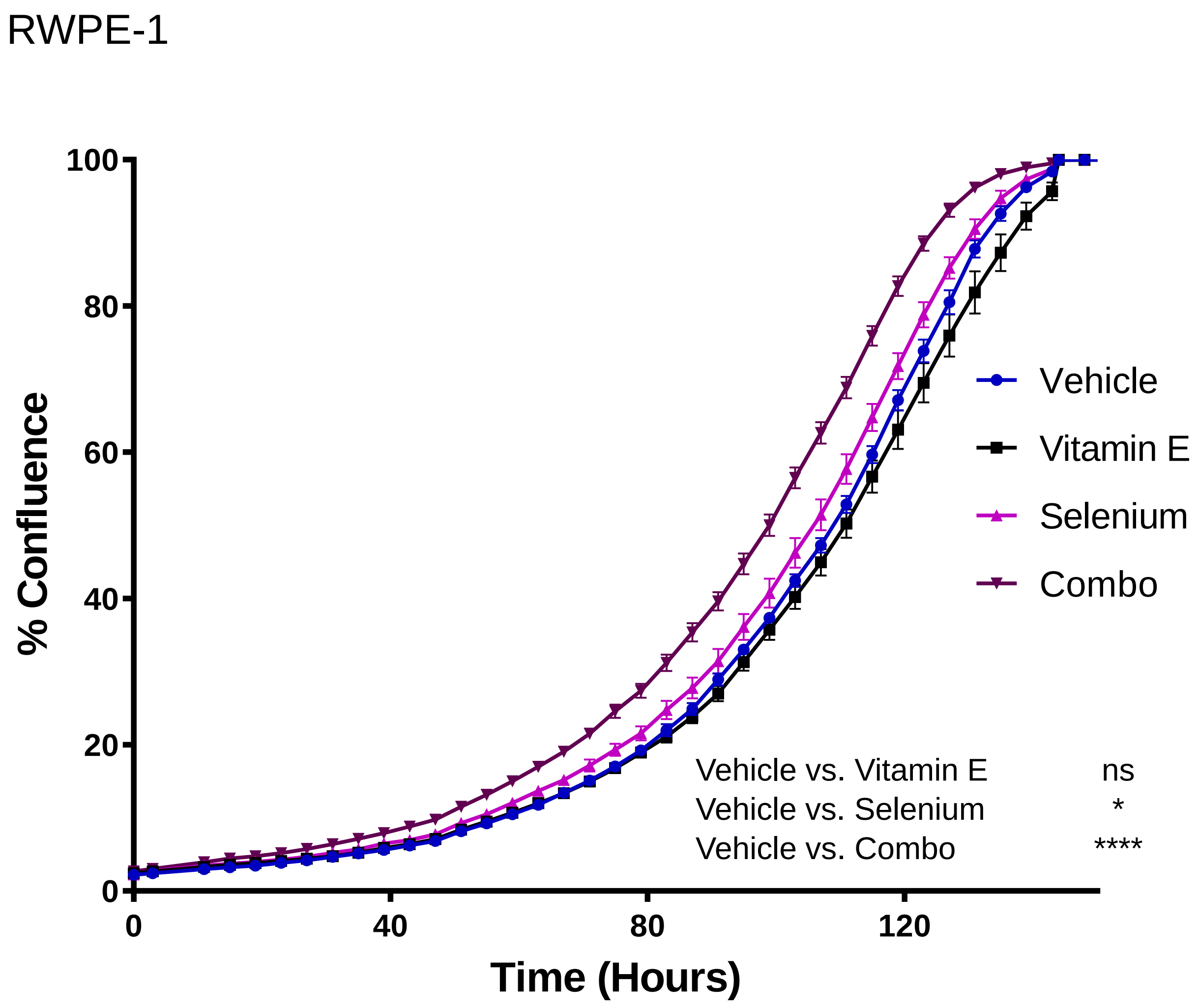

Supplementary Figure S2

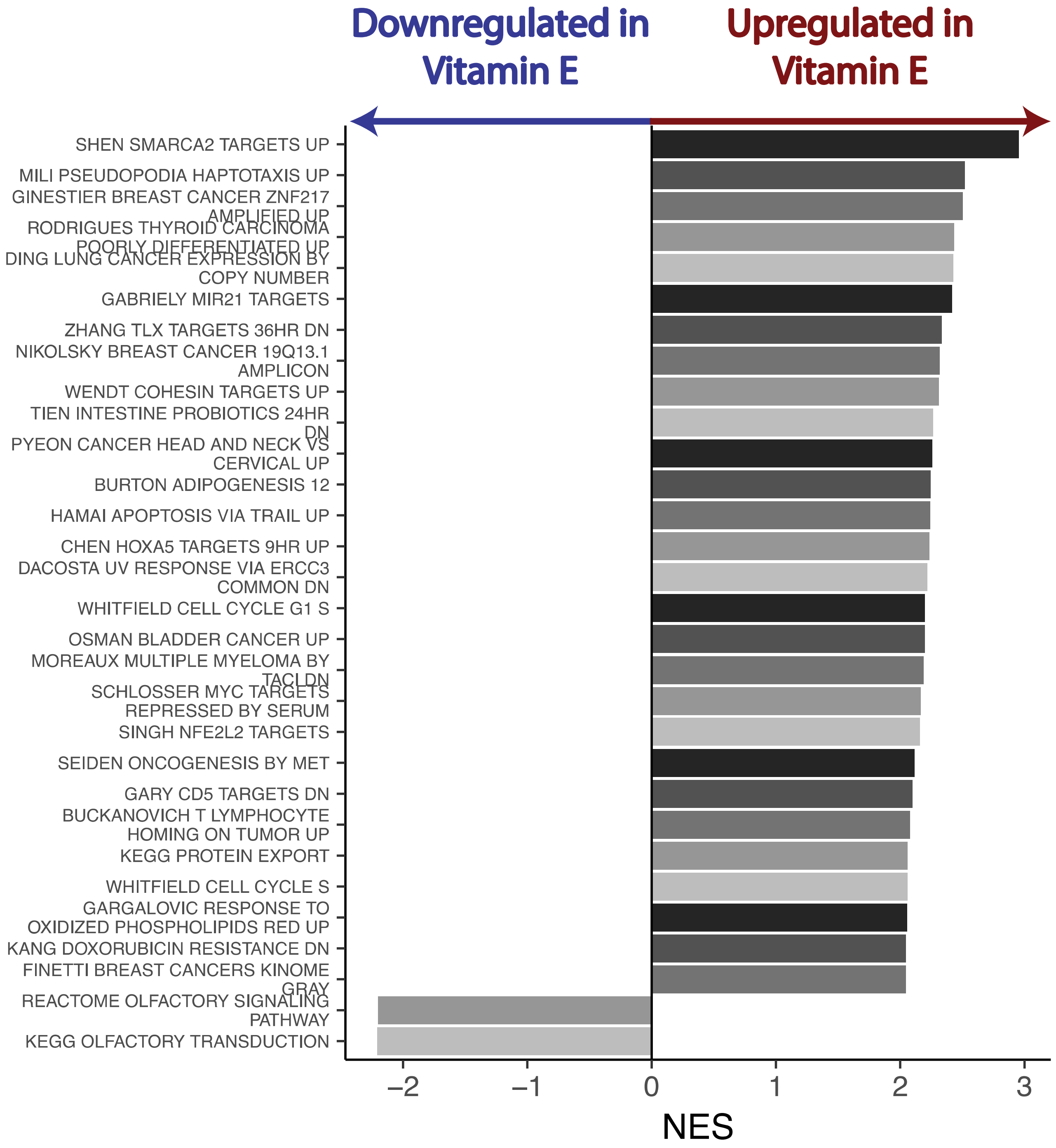

Supplementary Figure S3

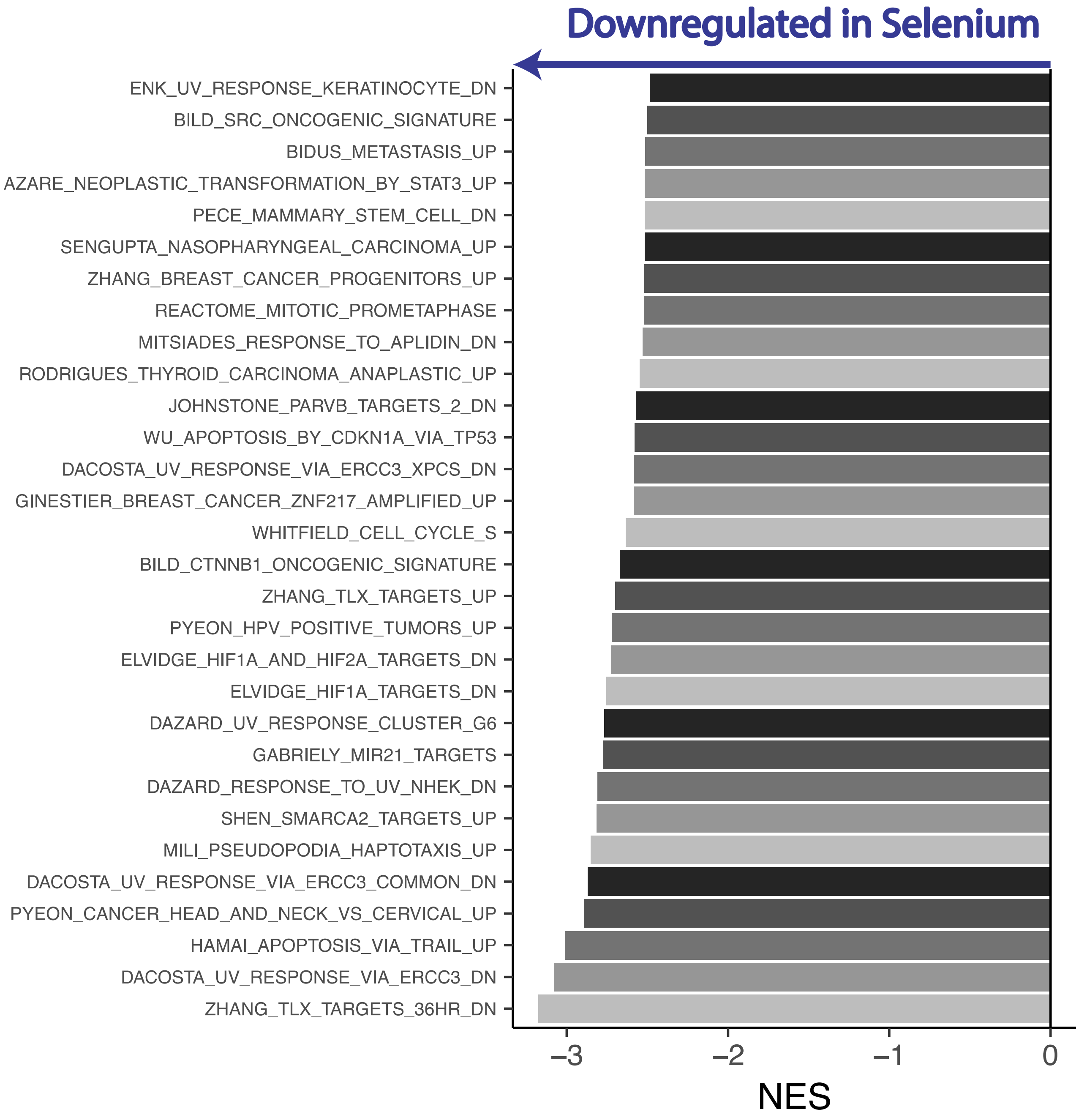

Supplementary Figure S4

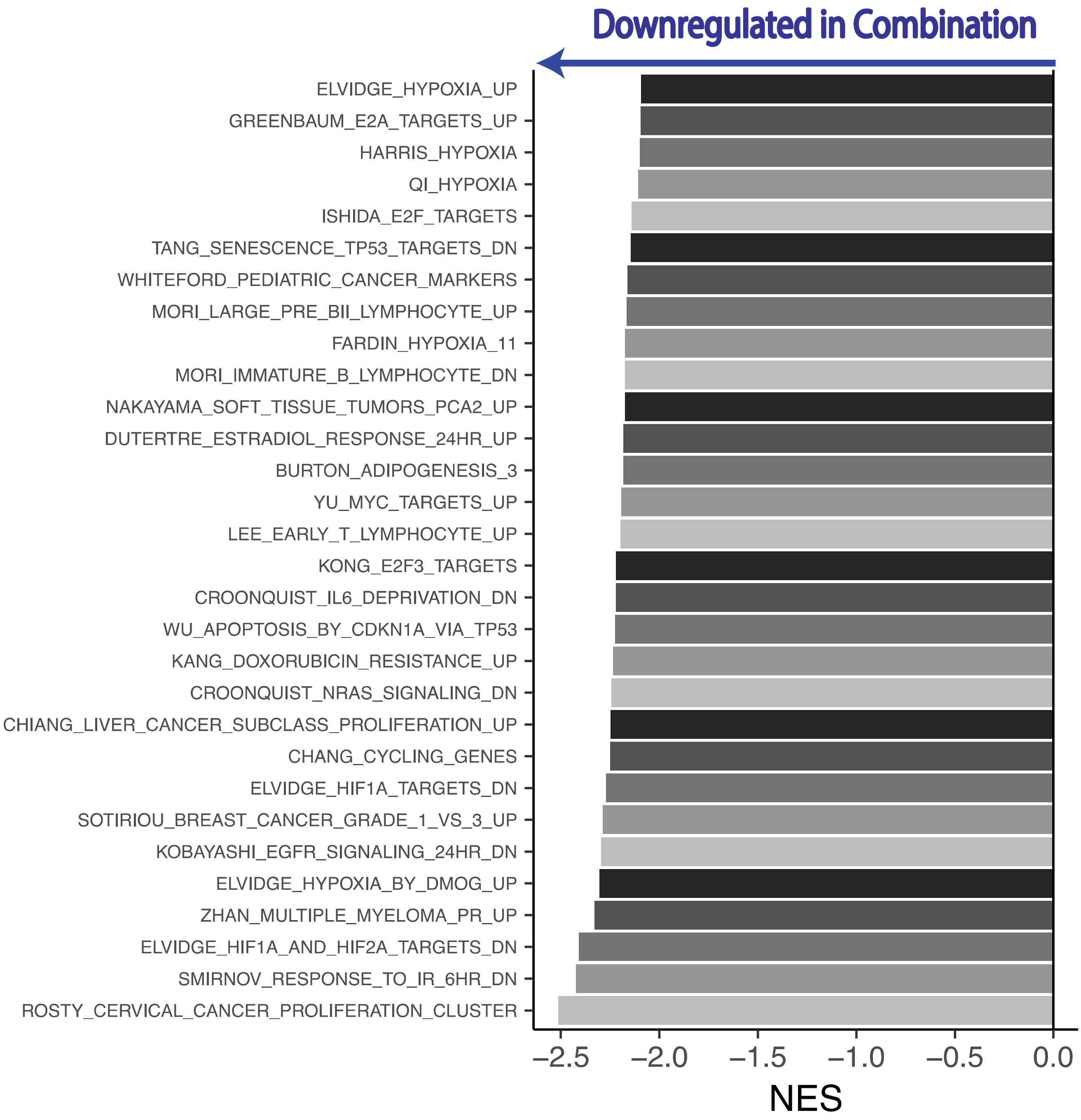

Supplementary Figure S5

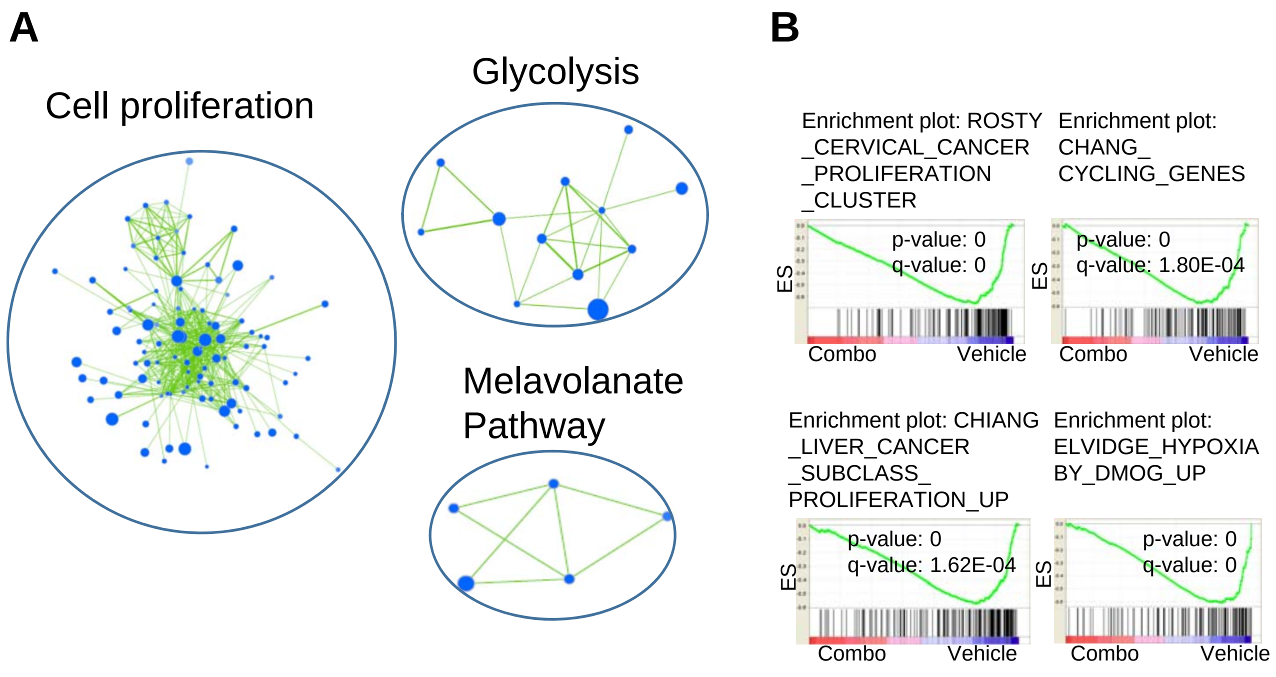

Supplementary Figure S6

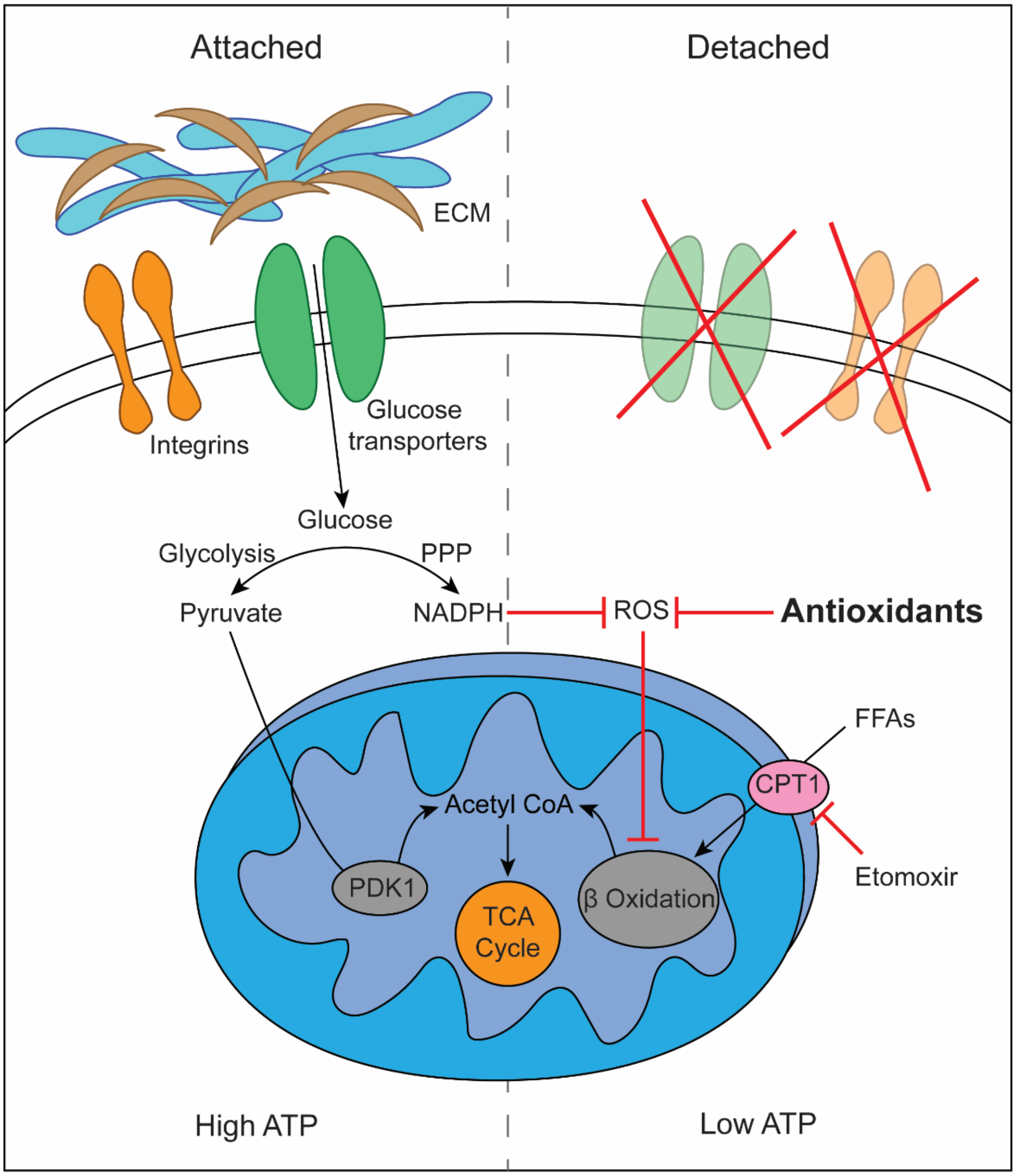

Supplement: Supplementary file 1 — Supplementary Information [file 41598_2017_16459_MOESM1_ESM.pdf]
